# Patient-centered clinical decision support challenges and opportunities identified from workflow execution models

**DOI:** 10.1093/jamia/ocae138

**Published:** 2024-06-22

**Authors:** Dean F Sittig, Aziz Boxwala, Adam Wright, Courtney Zott, Nicole A Gauthreaux, James Swiger, Edwin A Lomotan, Prashila Dullabh

**Affiliations:** Department of Clinical and Health Informatics, University of Texas Health Science Center, Houston, TX 77030, United States; Elimu Informatics, El Cerrito, CA 94530, United States; Department of Biomedical Informatics, Vanderbilt University Medical Center, Nashville, TN 37232, United States; NORC at the University of Chicago, Bethesda, MD 20814, United States; NORC at the University of Chicago, Bethesda, MD 20814, United States; Center for Evidence and Practice Improvement, Agency for Healthcare Research and Quality, Rockville, MD 20857, United States; Center for Evidence and Practice Improvement, Agency for Healthcare Research and Quality, Rockville, MD 20857, United States; NORC at the University of Chicago, Bethesda, MD 20814, United States

**Keywords:** decision support systems, clinical, workflow, patient-reported outcome measures, patient-generated health data

## Abstract

**Objective:**

To use workflow execution models to highlight new considerations for patient-centered clinical decision support policies (PC CDS), processes, procedures, technology, and expertise required to support new workflows.

**Methods:**

To generate and refine models, we used (1) targeted literature reviews; (2) key informant interviews with 6 external PC CDS experts; (3) model refinement based on authors’ experience; and (4) validation of the models by a 26-member steering committee.

**Results and Discussion:**

We identified 7 major issues that provide significant challenges and opportunities for healthcare systems, researchers, administrators, and health IT and app developers. Overcoming these challenges presents opportunities for new or modified policies, processes, procedures, technology, and expertise to: (1) Ensure patient-generated health data (PGHD), including patient-reported outcomes (PROs), are documented, reviewed, and managed by appropriately trained clinicians, between visits and after regular working hours. (2) Educate patients to use connected medical devices and handle technical issues. (3) Facilitate collection and incorporation of PGHD, PROs, patient preferences, and social determinants of health into existing electronic health records. (4) Troubleshoot erroneous data received from devices. (5) Develop dashboards to display longitudinal patient-reported data. (6) Provide reimbursement to support new models of care. (7) Support patient engagement with remote devices.

**Conclusion:**

Several new policies, processes, technologies, and expertise are required to ensure safe and effective implementation and use of PC CDS. As we gain more experience implementing and working with PC CDS, we should be able to begin realizing the long-term positive impact on patient health that the patient-centered movement in healthcare promises.

## Introduction

Traditional clinical decision support (CDS) focuses on providing clinicians with patient-specific preventive, diagnostic, treatment, or management guidance to help them provide the highest quality, safest care possible. Recently, there has been a push toward patient-centered clinical decision support (PC CDS) that exists on a continuum reflecting the degree to which CDS interventions (a) are based on patient-centered outcomes research (PCOR) findings, (b) incorporate patient-generated health data (PGHD)^1^ including patient-reported outcomes (PROs),^2^ patient preferences, or social determinants of health (SDOH), (c) are delivered directly to patients/caregivers via apps or portals, or (d) support shared decision-making.^3^ As with traditional CDS, some PC CDS interventions have shown positive outcomes, but gaps in our understanding of the sociotechnical factors that influence PC CDS design, development, implementation, use, and evaluation currently limit them from reaching their full potential.

The goal of this aticle is to use workflow execution models^4^ to highlight new considerations for PC CDS policies and procedures that healthcare systems, clinicians, electronic health record (EHR) developers, app developers, and others need to develop to support new and evolving workflows. Similar workflow models have been used successfully to develop guidelines for health information technology design in chronic care, for example.^5^ In this manuscript, we describe the use of newly created workflow execution models to explore 3 illustrative use cases for PC CDS: (**1) collection and use of patient-reported outcomes (PROs) data,** which are reports from patients about their health, quality of life, or functional status associated with the health care or treatment they have received; (2) **collection and use of patient-generated health data (PGHD) other than PROs** such as physiologic data from devices and wearables to improve the patient context; and (3) **encouraging or facilitating a shared decision-making discussion** where clinicians and patients make decisions together using the best available evidence. For each of these use cases, we explored the processes and tasks that must be accomplished by humans, computer applications, or a combination of the 2, to deliver high-quality, meaningful health outcomes.

## Background

PC CDS developers and evaluators can use workflow execution models to track, measure, and monitor the necessary data, information, and knowledge as the PC CDS moves through the clinical decision support phase of the PC CDS lifecycle.^6^^,^^7^ Such models are designed to highlight the possibilities, as well as the limitations, of our current understanding of the complex PC CDS space. For a PC CDS intervention to have the desired impact on either the healthcare delivery system or patient health, each model component must be designed and built to address patient and clinician needs, function as designed, and be used as expected. As the healthcare delivery system moves toward a more computer-enabled workflow system, it becomes more important to include the tasks and processes that the computer, which must be designed and developed, can do. These computer-enabled tasks support or replace tasks previously performed by clinicians or in some cases represent new tasks necessary to create new healthcare delivery processes, such as PC CDS. These computer-enabled tasks represent new work and new workflows for clinicians. The overarching goal of workflow execution models is to take into consideration the potential methods and sequencing of generating, sending, receiving, and acting on PC CDS interventions.

## Methods

We developed the workflow execution models using 4 methods: (1) a targeted literature review; (2) key informant interviews with 6 external CDS experts; (3) model refinement based on authors’ experience; and (4) validation of the models by a 26-member steering committee.

### Literature review

In several cases, we found the beginnings of prototype workflow execution models for each of the 3 types of PC CDS (CDS based on PRO data, CDS based on PGHD, and CDS that led to shared decision-making) in the literature.^8–10^ We iteratively augmented these early working model prototypes by adding in key workflow steps based on descriptions of the activities of people, the content of policies, the sequence of processes, and the steps in procedures reported in other publications that focused on specific PC CDS intervention implementation or evaluations.^11–13^ We stopped searching for additional workflow steps to add to our models when we were no longer identifying new steps (ie, often called saturation in qualitative research).

### Key informant discussions

We conducted 6 key informant interviews with 3 clinicians experienced in using PC CDS, 2 informaticians responsible for the design, development, implementation, and evaluation of PC CDS interventions, and 1 device and remote monitoring platform developer. The key informant discussions used early iterations of the workflow execution models to help focus the discussion on descriptions of current practices, policies, and approaches being used by healthcare systems to receive, curate, and manage PGHD data including PROs. Key informant discussions focused on challenges and gaps with current policies, or process activities.

### Model refinement

The authors of this article (A.B., P.D., E.A.L., D.S., J.S., A.W.) have extensive experience in designing, developing, implementing and using various PC CDS interventions and applications. The authors conducted several virtual meetings to brainstorm, iteratively refine, and reorganize the workflow models and identify challenges to and opportunities for future success.

### Steering committee input

After we reached consensus on the content of the models, the sequence of actions, and their visual display, we shared our models and findings with the AHRQ-funded Clinical Decision Support Innovation Collaborative (CDSiC) project steering committee which is a 26-member multi-stakeholder group that includes representatives from federal agencies, academic medical centers, informaticians, health information technology vendors, patient advocates, researchers/research organizations, and health systems. These external experts reviewed each of the models and compared the descriptions of the steps along with the sequence of steps outlined, to their own experiences in designing, developing, implementing, and using similar patient-centered clinical decision support interventions. When necessary, additional refinements to the description or sequence of steps in the models were made.

## Results

We identified the following 3 workflow execution models: collection and use of PROs, collection and use of PGHD other than PROs, and identification of opportunities for shared clinical decision making.

### Model 1: collection and use of PROs

**Figure 1.**
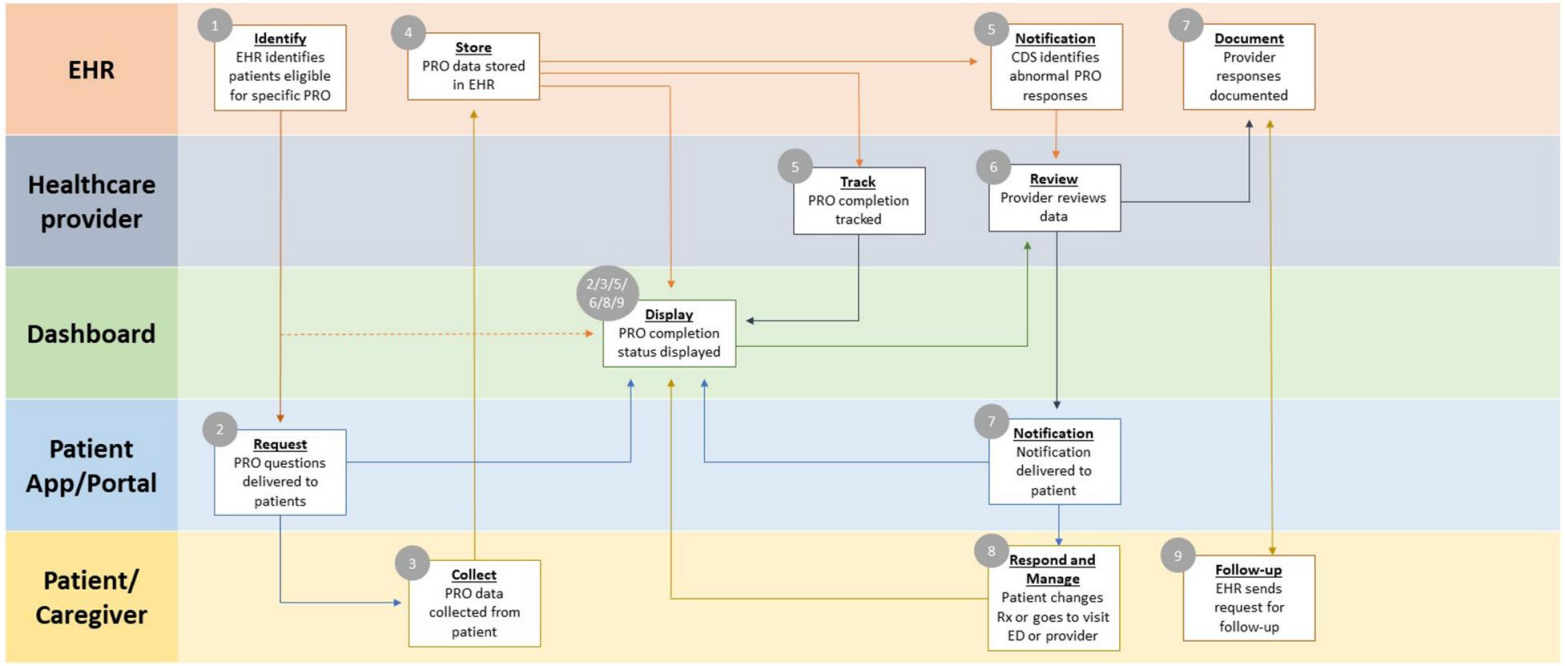
Illustration of a complete, idealized set of relationships and a preferred sequence of processes and tasks between patients, clinicians, EHRs, dashboards, and patient portals or apps for PROs. The process begins in the upper left-hand corner of the model below. In many cases, there are tasks or processes that are performed in parallel (ie, at or near the same point in time) by different actors (either humans or computer applications).

Table 1 lists the various workflow activities and actors involved in a hypothetical PC CDS depression screening example that is based on PROs along with example measures that could be used to assess each activity. In this scenario, the eligible patients receive the Patient Health Questionnaire (PHQ-9), which is a 9-question screening tool for depression. All asymptomatic adults 19 years or older who do not have a diagnosed mental health disorder or recognizable signs or symptoms of depression or suicide risk are eligible to be screened.^14^ The use of a self-administered questionnaire helps ensure that the eligibility screening process is free from bias (See Figure 1).

**Table 1.** Core activities of both humans and computer applications within the PRO workflow.^15^^,^^16^

Workflow activity	Definition	Actors involved	Depression screening workflow	Example measures
Identify	EHR identifies patients eligible for specific PROs (Human or CDS logic)	EHR to Patient App/Portal	Identify patients at risk for depression or those without recent screening in clinics with staff available to help.	Number of patients invited to participate Percentage of patients invited who accepted
Request	Deliver the PRO questionnaire to the patient	Patient App/Portal to Patient/Caregiver	Automated, 7 days before the appointment, based on an algorithm of last ePHQ-9 completion date. Patient receives a generic request to complete the ePHQ-9 and 2 reminders.	Number of PRO questionnaires sent Number of reminders sent Percentage of patients needing reminder
Collect	Patient completes and submits the PRO questionnaire	Patient/Caregiver to EHR	Patient completes the ePHQ-9 from home using patient portal or app, or in the doctor’s office using a patient kiosk.	Percentage of completed PRO questionnaires returned before visit Number of patients completing PRO questionnaires in office (kiosk) Percentage of patients completing PRO questionnaires via portal vs kiosk
Store	Store the PRO data in the EHR for future use	EHR to Dashboard and Healtcare provider	Patient’s responses to ePHQ-9 stored in their EHR.	Number of responses for each patient to ePHQ-9 Median number of responses for each patient Total computer storage area consumed by ePHQ-9 data
Track	Monitor the completion status of PRO questionnaire	Healthcare provider to Dashboard	ePHQ-9 completion status is tracked via appointment check-in or rooming views of EHR.	Percentage of patients who completed PRO before visit
Display	Create a visual display or graphical representation of the completion status data	Dashboard to Healthcare provider	Healthcare provider tracks completion rate, distribution, and individual ePHQ-9 scores.	Percentage of clinicians who reviewed dashboard before or at visit Percentage of patients in each depression severity category
Notification	Trigger CDS logic to compare responses to alert criteria and notify the patient	EHR to Healthcare provider and Patient App/Portal to Dashboard	Clinician (depending on problem notify patient as well) notified that a normal or a dangerous situation exists.	Percentage of patients generating an abnormal notification (alert)
Review	Clinical teams access and view PRO scores	Healthcare provider to Patient app/Portal	Clinician reviews ePHQ-9 before or during visit.	Elapsed time between PRO completion and data review Percentage of clinicians who reviewed PRO data before or at visit
Respond and Manage	Patient reviews data and plans, changes Rx, or visits provider	Patient/Caregiver to Dashboard	Patient encouraged to see provider/referral and/or start medication.	Percentage of patients encouraged to change medications Percentage of patients encouraged to see provider Percentage of patients referred to a specialist
Document	Archive the PRO scores for future use or use by other stakeholders	Healthcare provider and Patient/Caregiver to EHR	Clinic staff or clinician saves (ie, “files”) ePHQ-9 data to EHR.	Number of patients with PRO data saved Elapsed time between receipt of PRO data and completion of documentation
Follow-up	Follow-up periodically or after positive screening result	Patient/Caregiver to EHR	Healthcare organization tracks clinical and patient-centered outcomes.	Use appropriate patient-reported outcome measures associated with screening target Assess satisfaction with screening system

Abbreviations: CDS = clinical decision support; ED = emergency department; EHR = electronic health record; ePHQ-9 = electronic Patient Health Questionnaire-9; PHQ-9 = Patient Health Questionnaire with 9 questions; PRO = patient-reported outcome.

### Model 2: collection and use of PGHD

**Figure 2.**
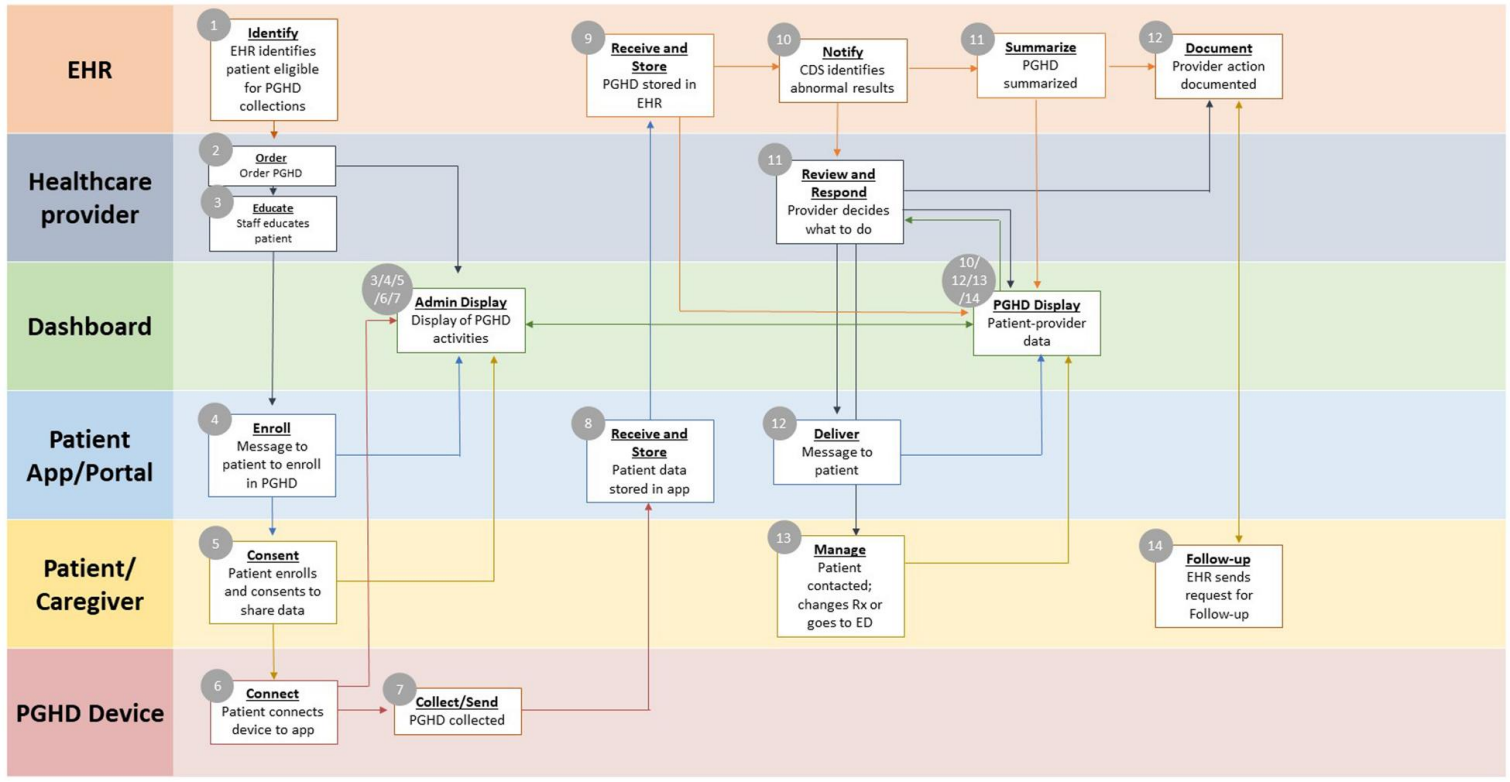
Description of a complete, idealized set of relationships and a preferred sequence of processes and tasks between patients, clinicians, EHRs, dashboards, PGHD devices, and patient portals or apps for collecting and utilizing PGHD. The process begins in the upper left-hand corner of the model below. In many cases, some tasks or processes are performed in parallel (ie, at or near the same point in time) by different actors (either humans or computer applications).

The second model involves PGHD that is used to drive specific PC CDS interventions. Table 2 provides an overview of the workflow components necessary to collect and use PGHD in the CDS process along with example measures that could be used to assess each activity. This example focuses on PGHD from a patient-controlled medical device, ie, a home blood pressure cuff. This information has been solicited and subsequently stored automatically in the healthcare organization’s EHR or some external database. We are deliberately not focusing on PGHD that are unsolicited by the clinician or their organizations. Unsolicited PGHD may arrive in the clinician’s in-basket or email in the event that a patient simply sends some physiologic measurements as free text in an email message or via an email attachment. While this unsolicited PGHD can be helpful to the clinician, it cannot be used by existing CDS interventions since it is not structured or easily stored in a structured manner within the EHR (See Figure 2).

**Table 2.** Core activities of both humans and computer applications in the PGHD workflow.^10^

Workflow activity	Definition	Actors involved	Remote BP monitoring for hypertension	Example measures
Identify	EHR identifies patients eligible for specific PGHD (Human or CDS logic)	EHR to Healthcare provider	Identify patients at risk for hypertension who might benefit from monitoring.	Number of patients identified Percentage of patients identified who clinician invited
Order	Clinician orders PGHD data collection for specific patients^13^	Healthcare provider to Dashboard	Patient receives an invitation to enroll in a BP monitoring program.	Number of patients receiving invitations to enroll Demographic breakdown of patients invited (eg, age, gender, ethnicity, SDOH)
Educate	Educate patient on how to use device safely and correctly	Healthcare provider to Patient/Caregiver	Care manager instructs patient on proper use of the device.	Number of patients educated Percentage of patients with device who got educated Patient satisfaction with education
Enroll	Request for patient to enroll in data collection program	Healthcare provider to Patient App/Portal	Clinician recommends home BP monitoring.	Number of patients sent request Measure of equitability in requests sent
Consent	Patient agrees to participate in data collection program	Patient/Caregiver to PGHD Device and Dashboard	Patient agrees to share BP data.	Number of patients who successfully consented to be in study Time between getting invitation and consenting to study
Connect	Patient connects device to App/Portal	PGHD Device to Dashboard	Patient enables data sharing between device and App/Portal	Number of patients who successfully connected device
Admin Display	Display activities related to PGHD collection	Dashboard	Activities displayed on dashboard.	% of clinicians responsible for a patient who review dashboard; % of patients reporting PGHD who review dashboard
Collect/Send	Patient uses device to collect and send physiologic data to the Patient App which sends it to their EHR	PGHD Device to Patient App/Portal	Patient begins collecting BP data. BP data are sent to clinician.	Number of patients who report collecting data Number of patients sending data Number of times each patient sent data Length of time between first and last submission of data
Receive and Store	Receive data from patient and store in the app and EHR	Patient App/Portal to EHR	Clinician receives data from patient.	Number of patients from whom data was received Number of times data was received Amount of data received in mb
Notify	Notify clinician/care team and patients of potentially dangerous findings and potential action steps	EHR to Healthcare provider	Clinician/patient notified re: abnormal findings.	Number of clinicians notified Percentage of patients sending data that generated a notification
Summarize	Analyze and summarize patient data	EHR to Dashboard	BP values and trends are summarized and checked for potentially life-threatening abnormalities or changes.	Mean time required to analyze/summarize data
PGHD Display	Display patient data for patients and clinician	Dashboard to Healthcare provider	Patient data displayed on dashboard.	User perceptions of display quality, accuracy, usefulness, etc.
Review and Respond	Review patient data	Healthcare provider to Patient App/Portal and Patient/Caregiver	Patient and provider review patient data.	Percentage of patients with data that were reviewed within x days
Deliver	Deliver normal and abnormal findings to patient	Patient App/Portal to Patient/Caregiver	Clinicians call, text, or email patients (depending on severity of symptoms) with instructions on how to manage BP changes (eg, increase dose of medication or come to the ED).	Number of messages sent Mean time between receipt and reading of message Mean time to respond to emergency notification
Manage	Patient reviews data and plans changes Rx or visits provider	Patient/Caregiver to Dashboard	At some point, the clinician needs to manage the patient’s condition via changes to medications, or lifestyle counselling, for example.	Percentage of patients contacted at least 1 time in first x months Mean number of times patients were contacted in x months Type of management actions taken
Document	Store raw data values, summaries, and follow up actions in patient’s EHR or external DB linked to patient ID	Healthcare provider to EHR	A summary of the patient’s BP readings and clinician instructions for changes in medication management are stored in the EHR.	Number of patients managed in time period Number of changes in therapy made Number of patient visits
Follow-up	Follow-up periodically or after completion of time- or condition-limited monitoring	Patient/Caregiver to EHR	Healthcare organization tracks clinical and patient-centered outcomes.	Use appropriate patient-reported measures of monitoring target Assess satisfaction with monitoring system

Abbreviations: BP = blood pressure; CDS = clinical decision support; DB = data base; ED = emergency department; EHR = electronic health record; mb = megabytes; PGHD = patient-generated health data.

### Model 3: shared decision-making

**Figure 3.**
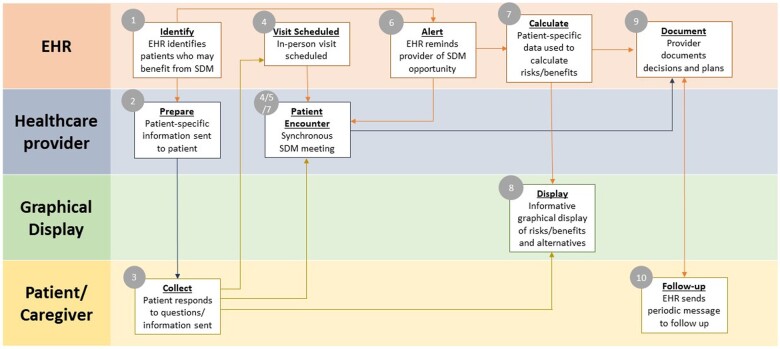
Illustration of a process which begins in the upper left-hand corner of the model below. In many cases, there are tasks or processes that are performed in parallel (ie, at or near the same point in time) by different actors (either humans or computer applications).

The third model focuses on shared decision-making, which involves a collaboration between patients and clinicians to arrive at healthcare decisions grounded in evidence, the expertise of the care team, and the patient's values, objectives, preferences, and individual circumstances.^17^ While patient participation in the clinical decision-making process is being increasingly promoted, many patient-related factors (eg, lack of medical knowledge, lack of confidence, co-morbidities, and other sociodemographic information) and clinician-related factors (eg, desire to maintain control, lack of time, personal beliefs, and lack of training in establishing and maintaining patient–caregiver relationships) make such interactions challenging.^18^ In an effort to facilitate shared decision-making between patients and clinicians, healthcare organizations have begun to develop CDS interventions that support shared decision-making processes. Table 3 provides an overview of the workflow components and actors necessary to implement shared decision-making in the healthcare delivery process along with example measures that could be used to assess each activity. For example, a patient can be sent easy-to-understand information prior to their visit to help them understand the basic clinical details of their condition. Data on patient’s needs, preferences, and goals can also be elicited from the patient either before or during the visit. This information can then be visualized and compared to reference ranges, if applicable, to help illustrate the options and potential outcomes and tradeoffs associated with selecting among different treatment options.^19^ In this way, PC CDS can be used both asynchronously or synchronously to facilitate the shared decision-making process (See Figure 3).

**Table 3.** Shared decision-making workflow elements.

Workflow activity	Definition	Actors involved	**Decision re: anticoagulation therapy** ^20^	Examples measures
Identify	EHR identifies patients who may benefit from SDM	EHR to Healthcare provider	Patients with atrial fibrillation who are not on anticoagulation therapy.	Number of patients identified as eligible to participate in SDM
Prepare	Patient-specific information sent to patients to help them prepare for visit	Healthcare provider to Patient/Caregiver	Patient receives easy-to-understand information about their condition and different treatment alternatives.	Number of patients sent information prior to visit
Collect patient needs, preferences, and goals	Patient responds to questionnaire or information sent or given to them	Patient/Caregiver to EHR, Healthcare provider, and Graphical display	Patient fills out questionnaire explaining their needs, preferences, and goals for treatment.	Number of patients responding to questionnaire
Visit scheduled	Patient and healthcare provider agree on date/time and place	EHR to Healthcare provider and Patient/Caregiver	Patient/Caregiver and healthcare provider agree to discuss alternatives.	Number of visits scheduled Number of no-shows
Patient Encounter	Patient/clinician synchronous meeting (virtual or in-person)	Healthcare provider and Patient/Caregiver to EHR	Patient arrives at clinic or joins remote teleconference.	Number of patient visits Demographic breakdown of patients (eg, age, gender, ethnicity, SDOH)
Alert	Reminder to clinician that shared decision-making opportunity exists	EHR to Healthcare provider	Clinician receives alert in EHR workflow before ordering anticoagulation therapy.	Percentage of alerts sent to clinicians Percentage of alerts that resulted in SDM discussion (Note: for a more involved and reliable measure, one could use the SDM-Q9^21^)
Calculate	Algorithm uses patient-specific EHR data coupled with patient preferences to create a personalized risk/benefit assessment for patient	EHR to Graphical display	Patient-specific risk assessment calculated based on existing EHR data; Assessment updated based on information gained during shared decision-making session.	Number of patient-specific assessments completed Percentage of patient-specific assessments updated during discussion Elapsed time to calculate patient-specific risk estimate
Display	Talking points for clinician/patient discussion, visual display, or risk calculator (optional)	Graphical display to Patient/Caregiver and Healthcare provider	Clinician reviews patient-specific risk/benefit assessment with patients and discusses alternatives.	Total time for SDM discussion Percentage of clinicians that share displayed data with patient
Document outcome	Document outcome of session	EHR and Healthcare provider	Clinician documents the results of discussion in EHR.	Percentage of patients who participated in a SDM session Type of action taken following SDM session
Follow-up	Follow-up on results of session	Patient/Caregiver to EHR	Healthcare organization tracks clinical and patient-centered outcomes.	Use appropriate patient-reported measures of SDM outcomes^22^ Assess decisional conflict and/or regret^23^

Abbreviations: EHR = electronic health record; SDOH = social determinants of health; SDM = shared decision-making.

## Discussion

Developing the PC CDS workflow execution models highlighted many new policies, processes, technologies, and expertise required to design, develop, implement, and use PC CDS safely and effectively within any healthcare organization. We identified these new aspects of PC CDS based on the authors’ collective experience, knowledge, and understanding of the sociotechnical constraints of implementing new, state-of-the-art health information technology interventions within complex healthcare delivery organizations. In addition, implementation of PC CDS adds additional sociotechnical constraints to previous clinician-focused CDS, such as the need to (1) extend the reach of the healthcare organization beyond the physical confines of the clinic, (2) interact with patients outside normal clinic working hours, (3) engage patients in clinical decision-making activities, and (4) support collection, documentation, and display of PGHD, including PROs from patients in support of patient-centered care. These 4 changes combine to place several new expectations on clinicians, healthcare delivery systems, payers, and technology companies.

Across the 3 illustrative use cases, we identified 7 major PC CDS-related challenges that must be addressed. Addressing these challenges provides significant opportunities and important considerations for the collection and use of new, patient-centric data that have implications for policies, procedures and operations for healthcare systems, researchers, administrators, health IT departments, and app developers to address. These include considerations around frequency of data collection, data validity, provenance, precision, accuracy, and reliability. Additional considerations focus on whether these are data that are solicited and requested by a health system and the processes they have in place to monitor and manage these data.

Next, we describe the 7 major PC CDS-related challenges and opportunities.

**1. Policies, procedures, and people are required to ensure PGHD including PROs are documented, reviewed, and managed by appropriately trained clinicians, between visits and especially after working hours.** The vast majority of a patient’s life occurs outside their infrequent, short interactions with the healthcare delivery system. The data collection components of PC CDS workflows bring patient data directly into the EHR, via manually entered or automatically collected physiologic data or from questionnaires soliciting patient-reported outcomes—requiring someone in the healthcare system to review and respond to critical information in an appropriate and timely manner. This will require new workflows regarding the data values that should trigger clinician notification and when and how these notifications should be made, data display options, and time to complete the new work. In addition, clinicians and healthcare delivery organizations are not currently prepared to handle the activities that should occur post-data collection, such as receiving and acting upon potentially life-threatening patient data between patient visits. To respond to this need, some healthcare organizations are starting to work with third-party remote patient-monitoring companies that offer services for patients with connected medical devices.^24^ These companies are developing digital platforms and hiring relevant clinical staff to monitor patient contributed data, a more encompassing term used for “data, information, or insights created, collected by, or originating from a person regarding his or her health or care.”^25^ Several of these companies have also developed the capability to exchange curated summaries of patient data with a healthcare system’s EHR.

**2. Policies, processes, and people are required to educate patients on how to use connected medical devices, and handle technical issues associated with them.** The number and variety of remote patient monitoring devices and PRO questionnaires available for clinicians to order and/or send to their patients is increasing rapidly. In addition, the sheer number of possible devices makes it difficult for healthcare organizations to assess, add to their formulary and orderable catalogs, and train clinicians on how to order them.^13^ Some younger, tech-savvy patients may not require special education on basic smartphone usage, downloading, configuring, and connecting new apps or physiologic measurement devices (eg, blood pressure cuff, pulmonary function testing equipment, or glucose monitoring). However, healthcare organizations will need staff with dedicated time to educate other patients on how to set up and use these devices to facilitate data collection activities. These may be as simple as how to log in to the device or how to enable sharing of data, or as complex as how to calibrate a pulmonary function testing device. Finally, as with any new technology, unintended consequences, technical issues, delayed adoption, and difficulty learning or adjusting to new ways of working will occur.^26^

In some cases, healthcare organizations are contracting with third-party remote patient monitoring companies that work directly with consumers to set up and manage technical issues that arise. To make the care transitions between the healthcare organization and the third-party companies as safe and seamless as possible, new policies on what setting changes can be made or suggested to the patients, especially those using a smartphone application, for example, will need to be developed. Often, these third-party remote patient monitoring companies establish agreements with the healthcare organization that specify the approved list of medical devices that can be ordered, supplied, and supported. Based on the approved list, these companies work directly with patients to set up and manage technical issues that arise with connected medical devices.

**3. Policies, processes, and standards are required to facilitate collection and incorporation of PGHD, including PROs, patient preferences, and SDOH into existing EHRs.** To date, EHR and app developers and patient-centered outcomes researchers have faced significant challenges in developing methods for collecting high-quality, error-free, patient-generated data and integrating it within existing EHR database structures. These challenges have included lack of agreement on what data should be collected from various devices (eg, raw data or summaries), where it should be stored, which data constitute the legal medical record, what standards should be used to encode the data, whether it should be integrated with clinician-collected data, and how its provenance should be recorded.^27^

In addition, processes and procedures within healthcare organizations can vary based on the type of device used. For example, some healthcare organizations require clinicians to review and approve patient-provided data before it can be integrated into the medical record. In other cases, if patients are enrolled into a care management program, such as for hypertension monitoring, data submitted by the patient does not require any additional review/adjudication before it is integrated into the EHR. The timing of data collection can also vary and present challenges. For example, some organizations encourage patients to complete PRO questionnaires prior to a scheduled visit, while others wait for the patient to arrive at the doctor’s office. Currently, efforts are underway to develop^28^ and/or encourage adoption of new controlled terminology standards including additions to ICD-10-CM,^29^ LOINC/SNOMED CT,^30^ and the Gravity Project.^31^

**4. Policies, procedures, and people are required to troubleshoot potentially erroneous data received from devices.** By definition, some percentage of automatically collected physiologic data will be erroneous. These erroneous data points may trigger CDS logic that sends a message to a clinician warning them falsely of a potentially life-threatening event. Other erroneous data may be in the normal range when the patient is, in fact, suffering a potentially life-threatening event. Either situation presents a potential liability for healthcare organizations and clinicians. Without a means to periodically test and fix these remote data collection devices, healthcare organizations are putting themselves in a precarious legal position and may not be able to respond in real-time to patient safety issues. The process for curating and adjudicating connected device data, including managing aberrant and potentially erroneous readings, is often included in the services remote patient-monitoring companies offer. In addition, several third-party remote monitoring companies are working on adding artificial intelligence capabilities into their data management platforms to look for anomalous trends in the raw patient data. Changes in these trends may signal erroneous data or an important change in the patient’s condition, both of which warrant additional attention and/or intervention.

**5. Dashboards within EHRs and patient portals are required to display longitudinal patient-reported data.** All of these new data will require new methods of integrating PGHD including PROs with existing EHR data while maintaining its provenance, displaying these data in a way that helps both clinicians and patients make the best decision, and developing metrics to summarize the data and use it for predictions.^32^ In some instances, the most important aspect of the data will be the changing values over time (ie, either improving or worsening). In others, the most recent value may be the most important. Currently, many organizations are in the process of designing and developing such PRO and PGHD longitudinal data displays.^32–34^ Several remote monitoring device companies are developing data visualization and analytic services alongside triaging services. As has been learned in many past experiments, the more patient and clinician input that goes into dashboard design, and the more closely integrated dashboard displays are with the EHR, the more likely the Patient App or Patient Portal will be useful and used.

**6. New, or revised, reimbursement policies are required to support these new models of care.** Collection, management, curation, and use of PGHD in support of patient-centered care will likely require new or revised reimbursement models from federal, state, and commercial payers. These new reimbursement policies will be especially important for ambulatory clinicians who are used to working a regular 5 day per week, 8-hour shift, while the new PC CDS-related care takes place 24-hours per day, 7 days per week. During the COVID-19 pandemic, for example, Medicare and Medicaid programs and commercial payers were forced to expand their telehealth reimbursement models to allow different communication applications (eg, Zoom, Webex, Teams) to be used, clinicians to work across state lines without additional medical licensure requirements, and clinicians to bill for their time as if it were an in-person visit.^35^ These changes may be a helpful blueprint for ways payment policy needs to change to prevent healthcare organizations from being forced to absorb these costs. The findings from our key informant discussions indicate that healthcare organizations are currently relying on billing for care coordination services to support PC CDS activities, but there are a significant number of resources and time that goes into working with new vendors and setting up processes and procedures that still goes unpaid. Without a reliable, payment model for these specific PC CDS activities, healthcare organizations will need to internally fund these activities.

**7. Strategies to support patient engagement with remote devices.** PC CDS interventions that rely on PGHD such as physiologic measurements and PROs will require sustained patient engagement in submitting data via remote devices, questionnaires, and other mechanisms. The findings from our key informant interviews indicate that keeping patients engaged in submitting data via remote devices has been a challenge for app developers and health systems alike. Specifically, patient use often declines after a few months. However, few studies have examined the factors that improve and sustain patient engagement, or what types of engagement lead to improved clinical or other outcomes.^3^ Informants mentioned one potential reason as the narrow focus on a specific clinical condition (eg, hypertension), when the reality is that patients often have multiple comorbidities and symptoms that they care about. Informants also mentioned that patients are increasingly accustomed to using the patient portal to interact with the health care system, since it is a more comprehensive source of information. Given this, patients may be less inclined to submit and view data via a separate device.

## Conclusion

The lessons learned from our delineation of workflow execution models for different types of PC CDS point out several new policies, processes, technologies, and people within healthcare delivery organizations, payers, and third-party remote patient-monitoring companies that must work together to design, develop, implement, and test these new interventions. As we gain more experience implementing and working with these new PC CDS-focused workflow execution models, identify the necessary internal and external resources, and gain critical buy-in from clinicians and patients, we should be able to begin realizing the long-term positive impact on patient health we all hope for.

## Data Availability

Not applicable.
